# A rare case of right lower quadrant pain

**DOI:** 10.1259/bjrcr.20170024

**Published:** 2018-10-31

**Authors:** Eduardo Teiga, Aleksandar Radosevic, Juan Sánchez, Marcos Busto, Guadalupe Aguilar, Jose Maiques, Daniel Flores, Ander Zugazaga, Javier Gimeno Beltran, Fernando Bazán

**Affiliations:** 1 Department of Radiology, IMI (Imagens Médicas Integradas) – Affidea Group, Lisbon, Portugal; 2 Department of Radiology, Hospital del Mar, Barcelona, Spain; 3 Department of Anatomical Pathology, Hospital del Mar, Barcelona, Spain; 4 Department of Interventional Radiology, Landspítali University Hospital, Reykjavíc, Iceland

## Abstract

Endometriosis of the appendix is a very rare entity and commonly affects females in childbearing age. Clinical presentation might be confusing varying from asymptomatic to acute abdominal pain and often mimicks acute appendicitis or chronic pelvic pain. Diagnosis is generally made after pathological examination as operative findings are usually non-specific. This condition poses a diagnostic challenge to radiologists and surgeons altogether and we therefore report a case of a middle aged female who presented with both right lower quadrant and right lower back pain. Recent literature is reviewed and radiological findings discussed.

## Clinical presentation

A 40-year-old female with no relevant medical history other than polycystic ovarian disease and resected endometrial polyps presented with both right lower quadrant and right lower back pain lasting for 2 days. No abdominal guarding was displayed at physical examination. Blumberg maneuver was negative. Blood work up tests were normal and transvaginal ultrasound ruled out gynecological disease.

## Investigations/IMAGING FINDINGS

**Figure 1.**
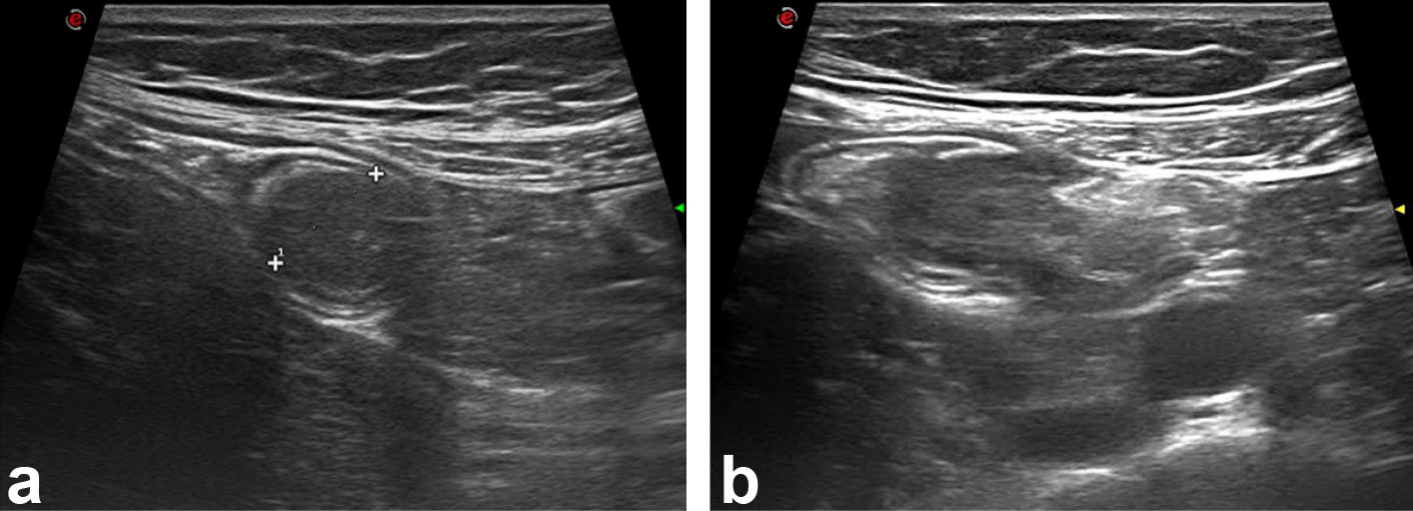
(a, b) Ultrasound revealed a homogenous hypoechoic and thick-walled tubular lesion with regular forms located at the pericecal area; no internal vascularity was noted.

**Figure 2.**
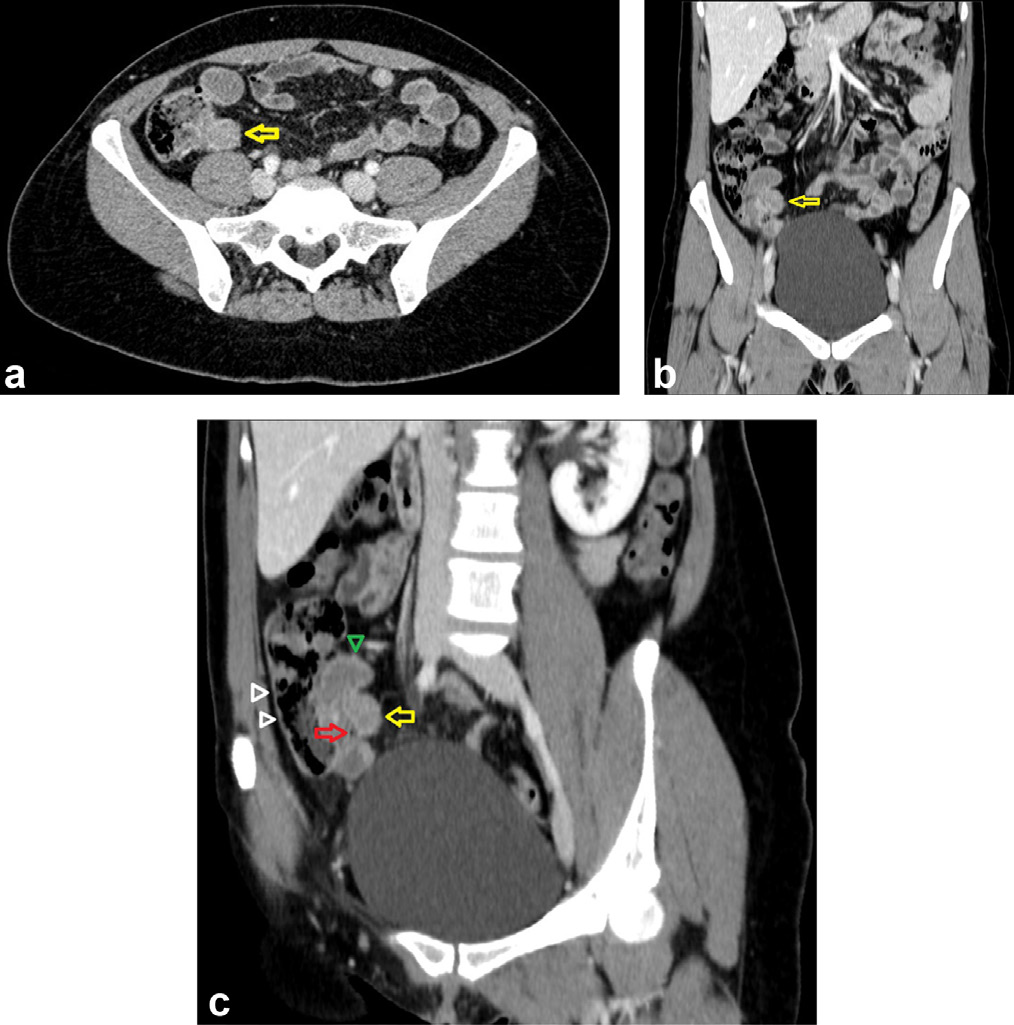
(a, b) CT examination depicted a non-specific focal mass (yellow arrow) in the distal third of the appendix without any evidence of inflammation. (c) Coronal modified view better depicts the relation of the appendiceal mass (yellow arrow) with the ileum (green arrowhead), appendix (red arrow pinpointing the air bubble in its lumen), and the cecum (white arrowheads).

**Figure 3.**
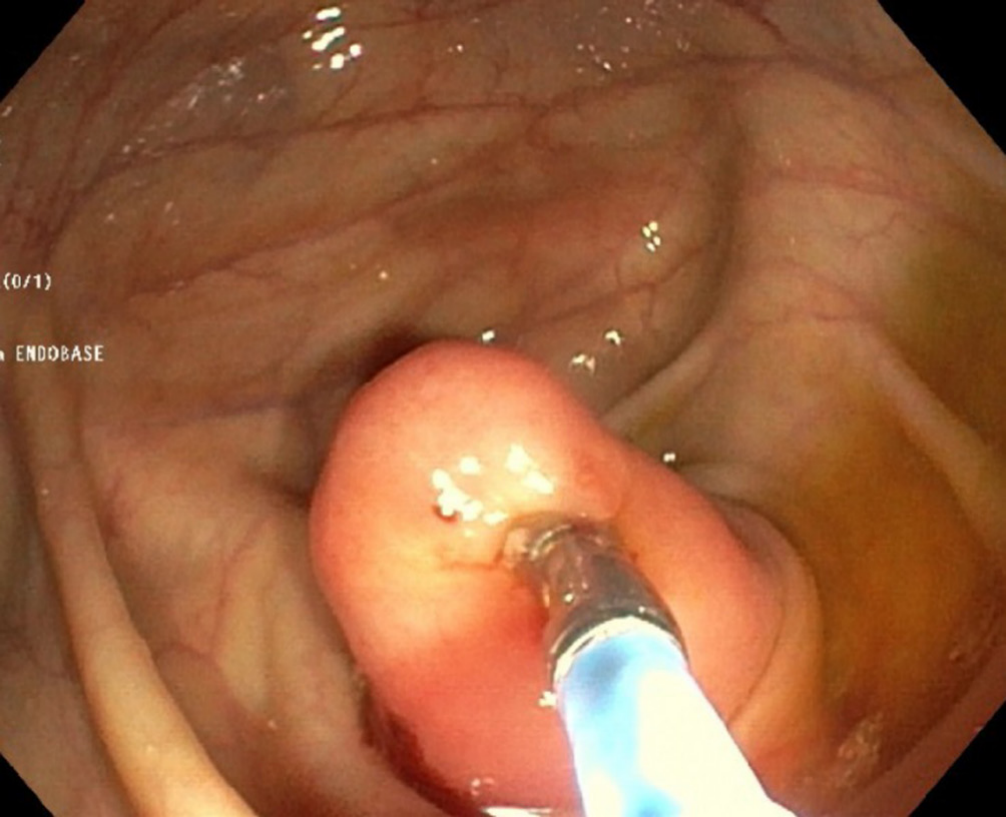
Endoscopy confirmed a 3 cm endoluminal protruding appendiceal mass displaying a hard consistency and an overlying smooth mucosa.

The ultrasound revealed a homogenous hypoechoic and thick-walled tubular lesion with regular forms located at the pericecal area; no internal vascularity was noted (Figure 1a,b). The CT examination depicted a non-specific focal mass (Figure 2a,b–yellow arrow) in the distal third of the appendix without any evidence of inflammation. Coronal modified view (Figure 2c) better depicts the relation of the appendiceal mass (yellow arrow) with the ileum (green arrowhead), appendix (red arrow pinpointing the air bubble in its lumen) and the cecum (white arrowheads). Endoscopy confirmed a 3 cm endoluminal protruding appendiceal mass displaying a hard consistency and an overlying smooth mucosa (Figure 3). Pathological examination of the fragments obtained by endoscopic biopsies was unfortunately inconclusive.

## Treatment

The patient was submitted to a partial resection of the cecum including the appendiceal mass. Pathological analysis of the specimen revealed the diagnosis of appendicular endometriosis.

## Outcome and follow-up

The patient was discharged 4 days after surgery and was gynecologically assessed afterwards. Follow-up MRI depicted two foci of endometriomas at the right ovary.

## Differential diagnosis

Other potential entities to consider include appendicitis and a tumor of the appendix.

## Discussion

Endometriosis is defined as the presence of ectopic endometrium-like tissue outside the mucosal lining of the uterus. It is usually seen while exploring for pelvic pain, pelvic masses or infertility. It is estimated to affect about 15% of the fertile and 50% of the infertile females. ^1,2^ Extragenital endometriosis is generally seen in the pelvic cavity and its location and extent considerably vary. The intestinal tract is frequently affected and the involvement might be intraluminal and/or extraluminal.

Intestinal endometriosis may clinically present as regional enteritis, appendicitis, ischaemic enteritis or colitis, diverticulitis or a neoplasm. Appendiceal endometriosis is rare; its incidence rate is less than 1% [^3^] and its clinical manifestations are usually not different from classical acute appendicitis.^1,3,4^ It may also present as intussusception, obstruction, lower intestinal bleeding and even perforation, especially during pregnancy.^4^ The operative findings are often not specific,^2^ the diagnosis being mostly made after pathological examination of the excised appendix. A long history of pain in the right lower quadrant of the abdomen in a female with an intermittent course, who has been known to have endometriosis is the sole clinical sign that can induce to suspect appendiceal endometriosis. However, one must remember that only 41% of these patients will complain of a “cyclic” right lower quadrant pain.^1^

Ultrasound is the first-line imaging technique for the evaluation of suspected endometriosis. However, evaluation is usually limited to the detection of endometriomas involving the ovary. The classic “chocolate cyst” appearance of an endometrioma is a homogeneous, hypoechoic lesion within the ovary with low-level echoes and no internal blood flow. Findings are however highly variable, with a spectrum of appearances related to degradation of blood products over time owing to the chronic nature of these lesions. Less common findings such as septations, a fluid–fluid level, a thickened wall, mural nodularity due to retracting clot, a solid mass, calcifications, and others overlap with entities such as hemorrhagic cysts, dermoids, and cystic ovarian neoplasms. MRI provides additional and more specific information not only in endometrioma characterization (with the classic hyperintensity on *T*_1_ weighted images and loss of signal—“shading”—on *T*_2 _weighted images), but also in pelvic involvement by endometriotic foci, being extremely useful in demonstrating pelvic distortion caused by fibrosis and adherences.^5^ One must bear in mind that the intestinal wall usually depicts as thickened, with low signal or density because of the hypertrophy of the muscular layer.^6^ MRI is helpful in differentiating endometriomas from dermoids. Dermoids may also appear hyperintense on *T*_1 _weighted images but contain fat. They will therefore display a signal intensity decrease on fat-suppressed images or show chemical shift artifact. CT plays a secondary role for the evaluation of a suspected endometrioma due to its non-specific imaging features generally appearing as complex cystic pelvic masses with high-density fluid components, findings commonly seen in hemorrhagic cysts and neoplasms. Alternatively, a discrete mass might not be visible and non-specific appendiceal dilatation without evidence of inflammation represents the only finding.^[Bibr b7]^ If endometrial involvement is isolated to the appendix, the imaging features of endometriosis with secondary appendicitis are most commonly indistinguishable from acute appendicitis. Other more typical findings such as complex adnexal cysts and hydrosalpinx may however suggest the diagnosis of endometriosis.

Laparoscopy is currently considered the gold standard for the diagnosis of endometriosis and careful examination of the abdominal cavity is performed in order to fully determine the extent of disease. Treatment consists primarily of surgery and is determined by the age of the patient and the severity of symptoms. Visual inspection alone might miss the foci of appendiceal endometriosis; incidental appendectomy is therefore recommended in patients with severe pain. Post-operative follow-up is mandatory.^8,9^

In conclusion, the present case highlights the radiological ancillary features of appendiceal endometriosis consisting of dilatation of the appendix and lack of inflammatory findings in a female who evoked no history of cyclic right lower quadrant pain. This rare condition is mostly diagnosed by histological examination following an appendicectomy performed for a different indication.

## Learning points

- Endometriosis of the appendix is a rare entity and its pre-operative diagnosis is extremely difficult.
- Consider its diagnosis in young females complaining of non-specific recurrent lower abdominal pain, especially when a history of infertility is present.
- Radiological ancillary features often consist of appendix dilation and lack of inflammatory findings.
- Histopathological examination provides its definitive diagnosis.
- Appendectomy simply cures the acute symptoms; gynecological follow-up after surgery is required for the underlying invasive endometriosis

**Figure 4.**
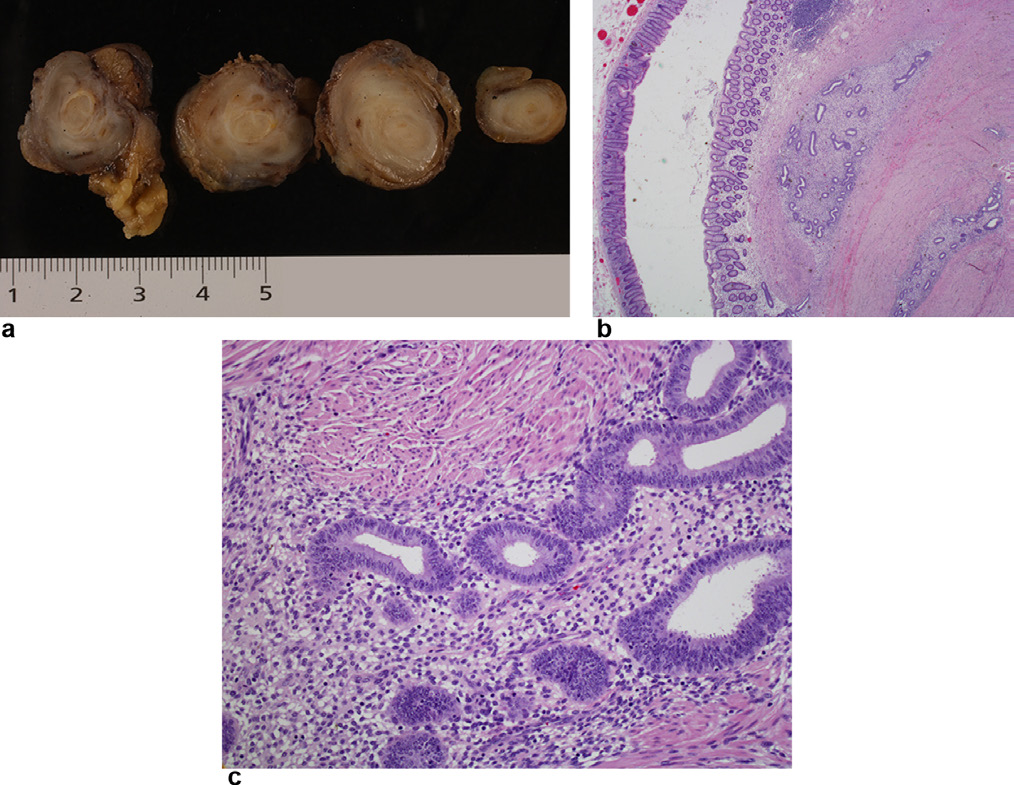
Histopathological examination (a) Gross examination of the appendectomy specimen. The wall of the appendix was diffusely and concentrically thickened, apparently due to the muscular layer and presenting yellowish areas of softer consistency. (b) Hematoxylin-eosin stain 2X: Low-power view of the wall of the appendix showing bland-like appearing glands surrounded by a fusocellular stroma, highly suspicious for endometriosis. (c) (HE, 20X): Detail of an endometriosic foci, containing cuboidal cells, some of them with cilia, without cytological atypia, reproducing proliferative benign endometrial mucosa.
